# The association between polymorphisms near *TMEM18* and the risk of obesity: a meta-analysis

**DOI:** 10.1186/s12920-021-01025-7

**Published:** 2021-07-06

**Authors:** Natalia Koj, Łukasz Grochowalski, Justyna Jarczak, Weronika Wójtowicz, Marta Sobalska-Kwapis, Marcin Słomka, Błażej Marciniak, Dominik Strapagiel

**Affiliations:** 1 Biobank Lab, Department of Molecular Biophysics, Faculty of Biology and Environmental Protection, University of Lodz, Lodz, Poland; 2 BBMRI.Pl Consortium, Wroclaw, Poland

**Keywords:** Obesity, TMEM18, Meta-analysis

## Abstract

**Background:**

Many studies have proposed that the pathogenesis of obesity has a genetic basis, with an important risk factor being the presence of polymorphisms in the region of the *TMEM18* gene, which plays a significant role in feeding behaviour; however, subsequent studies among different ethnic populations and age groups have shown inconsistent results. Therefore, this present meta-analysis examines the relationship between *TMEM18* polymorphisms with the risk of obesity with regard to age group and ethnic population.

**Methods:**

A literature database search was conducted for available relevant studies investigating the association between obesity risk and the presence of rs6548238, rs4854344, rs11127485, rs2867125 and rs7561317 polymorphisms in *TMEM18*. Pooled odds ratio (OR) and 95% confidence intervals (95% CI) were calculated by either a fixed-effects model or random effect model based on a heterogeneity test. The meta-analysis of rs6548238 and its surrogates examined the relationships between 53 395 obesity cases and 123 972 healthy controls from 27 studies and published data from the POPULOUS collection (Poland).

**Results:**

A significant association is observed between rs6548238 (and surrogate) and obesity risk, with OR = 1.25 (95% CI: 1.08–1.45). Regarding population type, a significant association was revealed among groups of Europeans with OR = 1.32 (1.10–1.59) and Mexicans with OR = 1.39 (1.13–1.73). However, a lack of statistical significance was noticed in groups in Asia with OR = 1.11 (95% CI: 0.86–1.42). Regarding age, a significant association was observed among children with OR = 1.28 (95% CI: 1.18–1.39) but not in adults OR = 1.21 (95% CI: 0.92–1.58).

**Conclusions:**

The polymorphisms near *TMEM18* appear to play a role in the development of obesity. Our findings indicate that differences exist between ethnic populations and age groups, supporting those of a previous study showing the various effects of genetic factors on age and ethnic groups.

**Supplementary Information:**

The online version contains supplementary material available at 10.1186/s12920-021-01025-7.

## Background

Obesity is a nutritional disorder associated with weight gain [1] caused by extended maintenance of positive energy balance, i.e. where the energy consumed is higher than energy expended [2]. This phenomenon leads to abnormal or excessive fat accumulation [3], which entails the number of mechanisms impairing health. Excessive body weight greatly increases the risk of chronic disease, including depression, type 2 diabetes, cardiovascular disease or certain types of cancer [4]. Unfortunately, 2016 World Health Organisation data indicates that over 650 million adults were obese worldwide, a figure that nearly tripled since 1975 [5].

Obesity is classified by Body Mass Index (BMI), an index of weight-for-height defined as weight in kilograms divided by the square of height in meters (kg/m^2^). In adults, the threshold for obesity is considered to be a BMI of 30 [5], while age and sex-specific BMI-for-age percentile curves are used to define obesity in children: the Center for Disease Control and Prevention defines obesity as BMI values greater than the 95th percentile for children of the same age and sex [6]. Obese children are likely to stay obese into adulthood and they more easily develop weight-related diseases at a younger age [7]. As human age may have a significant impact on the manifestation of obesity, many studies concerning obesity are presented with regard to a specific age group [8]. In addition, differences in the prevalence of obesity have also been observed across diverse ethnic groups; while these disparities may be caused by lifestyle, socioeconomic status and access to health care, they may be influenced by varying levels of biological susceptibility for obesity [9].

Obesity is considered by many researchers to be a heritable disease [10], and many polymorphisms in the human genome could contribute to its development. Understanding the genetic factors affecting obesity will allow easier identification of its cause [11]. Previous studies have found polymorphisms in human chromosome 2p25 to be associated with obesity [3]. The *TMEM18* gene, which is located at chromosome 2p25.3, encodes the transmembrane protein 18 (TMEM18), which is located in the nuclear membrane. This protein is reported to be involved in weight regulation through transcriptional repression [12]. TMEM18 is expressed in several regions of the brain, including the hypothalamus, which is responsible for the regulation of feeding behaviour. The relationship between *TMEM18* and changes in BMI has been confirmed by a third wave of GWAS (Genome-wide association study) [11], and subsequently confirmed by two independent follow-up studies [13, 14]. Apart from its involvement in obesity, TMEM18 may also influence cell migration by regulating neuronal stem cell mobility [15].

With the discovery of an apparent relationship between the *TMEM18* region and obesity, there is a clear need to determine more precisely the effect size of polymorphisms rs6548238, rs4854344, rs11127485, rs2867125, and rs7561317 localised near the gene itself with regard to age and population type. Therefore, the present meta-analysis reviews a range of data meeting the required criteria, with the age categorization.

## Methods

### Material and search strategy

Identification of relevant studies concerning the association of rs6548238 rs4854344, rs11127485, rs2867125, and rs7561317 polymorphisms with obesity was conducted by systematic literature search from PubMed, Science Direct, Web of Science databases and GWAS catalogue. The following search terms were used: obesity AND (TMEM18 OR GWAS). Obtained results were divided into two categories: age and population. No restriction was placed on the time period, sample size or language. If more than one article was published using the same case series, only the original study was included.

### Inclusion and exclusion criteria

The present meta-analysis included genetic association studies based on cohort or case–control models, in which the case subjects were obese and the control subjects were individuals within the normal BMI range. The following inclusion criteria were applied: (a) case–control or cohort studies presenting original data on the associations between the risk of obesity and TMEM18 polymorphisms (rs6548238 rs4854344, rs11127485, rs2867125 or rs7561317), (b) sufficient data for the calculation of odds ratio (OR) with the 95% confidence interval (95% CI) or calculated OR with 95% CI. The following exclusion criteria were applied: (a) review articles and meta-analysis, (b) studies on the association between other gene polymorphisms and obesity risks, (c) no information about number of alleles and number of cases and controls (d) studies based on non-human models (e) quantitative traits such as Body Mass Index or Body Fat Percentage since the data were not uniform (beta with 95% CI, mean with standard deviation or mean with 95% CI).

### Data extraction

Two researchers collected data independently. Disagreements were resolved by discussion with a third researcher. The following information was extracted from each study: (a) name of the first author, (b) year of publication, (c) country of origin, (d) ethnicity, (e) sample size, (f) association of the SNPs with obesity (OR and 95% CI), (g) analysed polymorphism, (h) number of case and controls, (i) mean age of case and controls.

### Description of additional dataset

Additional data from adults of Polish origin were collected between 2010–2012 within the TESTOPLEK research project and registered as the **POPULOUS** collection [16, 17] (**POPU**lation—**LO**dz **U**niver**S**ity Biobank) at the Biobank Lab of the Department of Molecular Biophysics, University of Lodz. The POPULOUS population collection is currently registered in Directory (v. 4.0) of BBMRI-ERIC consortium under the registration number bbmri-eric:ID:PL_BLUL:collection:POPULOUS_BLUL. POPULOUS contains saliva samples from over 10,000 volunteers from all over the Poland [18]. The participants, as well as the methods used for DNA extraction, quantification and genotyping are described elsewhere [17].

The approval for the present study was obtained from the University of Lodz Review Board (KBBN-UL/II/2014), and each participant from the POPULOUS study gave their written consent to take part. All procedures were performed in accordance with the Declaration of Helsinki (ethical principles for medical research involving human subjects) [17].

The BMI of each participant was calculated from the standard formula: BMI = weight/height^2^, based on self-assessment of height and weight controlled by trained pollsters. People were considered as obese when BMI values were ≥ 30, and were normal when BMI was in the range of 18.5–24.9 [19]. Allele frequency of additional data derived from POPULOUS collection was calculated using PLINK 1.07. Differences in the distributions of allele frequencies between normal weight and obese people were evaluated using an additive model.

The results indicate that 6047 unrelated Polish adult subjects were enrolled in the study group. Of these, 3 495 subjects remained after exclusion of underweight (BMI < 18.5) and overweight (BMI in the range of 25.0–29.9) individuals. Following this, 3 334 individuals, including 827 obese cases (BMI > 30) and 2667 control cases with normal weight (BMI in the range of 18.5–25.0), were successfully genotyped for SNPs rs6548238, rs2867125 and rs7561317. For rs4854344 there were 804 cases and 2 592 controls. 24 × 1 Infinium HTS Human Core Exome (Illumina Inc., San Diego, CA, USA) microarrays were used for genotyping according to the protocol provided by the manufacturer.

### Quality assessment

The Newcastle–Ottawa Scale [20] was used to assess the quality of studies included in the analysis. The analysis was conducted by two researchers independently, disagreements were resolved by discussion with a third researcher. The used methodology assesses studies in terms of Selection, Comparability and Exposure. The overall score is calculated in terms of stars awarded for each of nine questions. All studies included in the meta-analysis received at least seven stars (Additional file 8: Table S3), which suggested the high quality of the included publications.

### Data analysis

The association between obesity risk and the presence of rs6548238, rs4854344, rs11127485, rs2867125 or rs7561317 polymorphisms was estimated by calculation of pooled ORs and 95% CI in the allele model (C vs. T for rs6548238, T vs. G for rs4854344, for T vs. C for rs11127485, C vs. T, for rs2867125 or G vs. A for rs7561317). The combined outcomes were displayed as forest plots. Statistical differences of the pooled ORs were assessed using Z-test, *p* ≤ 0.05 was considered as statistically significant. The statistical analysis of the extracted data was conducted using Review Manager 5.3 [21]. This program was used to calculate the pooled effect size and sensitivity analysis and to assess the risk of publication bias.

Cochran’s Chi-squared test (based on Q test) was used to evaluate heterogeneity between studies, a *p*-value threshold of 0.1 was considered as statistically significant. The heterogeneity between the studies was also tested by I^2^ statistics; I^2^ values of at least 50% were taken as indicators of heterogeneity. When heterogeneity existed, a random effect model was used to evaluate the pooled ORs and 95% CIs, otherwise a fixed effect model was used.

Potential publication bias was assessed via visual inspection of the funnel plot, with an asymmetric funnel plot indicating publication bias. To identify the source of heterogeneity, a sensitivity analysis was applied by removing individual studies from the data for children or adults. Subgroup analysis was performed based on population type (European, Asian or Mexican) and age: adults (> 18 years) vs. children (≤ 18 years). A total of five polymorphisms were evaluated independently (rs6548238, rs4854344, rs11127485, rs2867125, rs7561317), as was rs6548238 and its surrogates.

The following studies were included in the meta-analysis: Albuquerque et al. [22], Almen et al. [23], Bradfield et al. [24], Cheung et al. [25], den Hoed et al. [26], Dusatkova et al. [27], Garcia-Solis et al. [28], Hong et al. [29], Hotta et al. [30], Leon-Mimila et al. [31], Liu et al. [32], Lv et al. [33], Ozdemir-Erdogan et al. [34], Rask-Andersen et al. [35], Rask-Andersen et al. [36], Rouskas et al. [37], Sandholt et al. [38], Speliotes et al. [13], Srivastava et al. [39], Thomsen et al. [40], Wang et al. [41], Willer et al. [11], Zhao et al. [42], Zhu et al. [43].

## Results

### Characteristics of the included individual studies

**Fig. 1:**
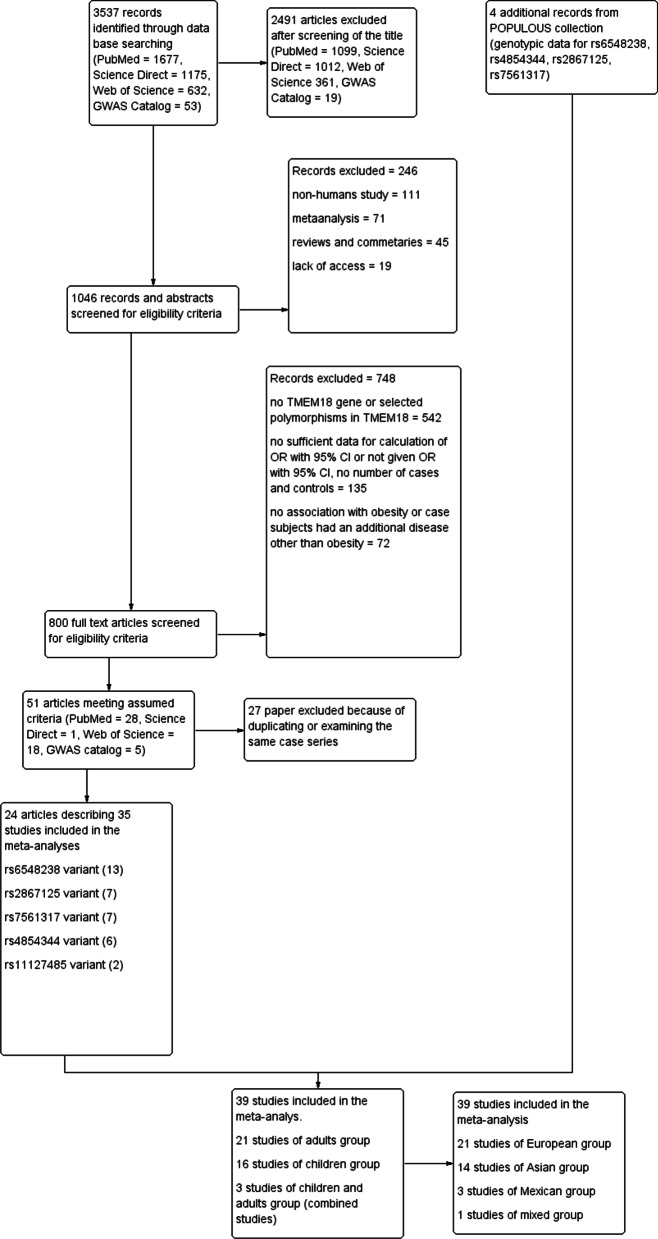
PRISMA flow diagram for the meta-analysis. Study selection process according to Preferred Reporting Items for Systematic Reviews and Meta-Analyses (PRISMA) guidelines. The PRISMA template is created within RevMan 5.3 software

The PRISMA (Preferred Reporting Items for Systematic Reviews and Meta-Analyses) flow chart explaining study selection and literature search is presented in Fig. 1. Searches of the databases using the keywords identified 3 537 potentially relevant reports. All articles were screened against the inclusion and exclusion criteria. After reading the titles, 2 491 irrelevant studies were excluded. Another 246 studies were excluded after reading the abstracts and 748 after reading the full texts. After applying all inclusion and exclusion criteria, 25 articles and 35 studies from the literature search and four additional studies from POPULOUS collection were analysed. Eight articles included more than one study [23, 30, 31, 35, 36, 39, 42, 44]. The characteristics of the included studies are shown in Additional file 6: Table S1. Regarding population type, 14 studies were performed on Asian populations, 21 on European populations, three on Mexican populations and one was mixed. Among these studies, 21 focused on adults (≥ 18 years), and 16 on children (< 18 years) and three were combined studies (children and adults together).

The sample size showed great variation, from n = 77 to n = 40 872. Throughout the meta-analysis, if the study contained results for more than one polymorphism, only rs6548238 was selected. Detailed information about the studies selected for the meta-analysis is gathered in Additional file 6: Table S1.

### Heterogeneity

The heterogeneity among studies was assessed by the Chi-squared test based on Q-test and the I^2^ statistics. The results of the I^2^ test found low heterogeneity concerning rs6548238 and its surrogate in the group of children (I^2^ = 54%, *p* = 0.01). Therefore the fixed effect model was used for further analysis. As the group of adults demonstrated high heterogeneity (I^2^ = 99%, *p* < 0.00001), the random effect model was used. Regarding population type, low heterogeneity was only observed in the Mexican group (I^2^ = 41%, *p* = 0.18); higher heterogeneity was observed among the European and Asian groups (I^2^ = 99%, *p* < 0.00001 and I^2^ = 83%, *p* < 0.00001 respectively).

### Quantitative data synthesis

**Fig. 2:**
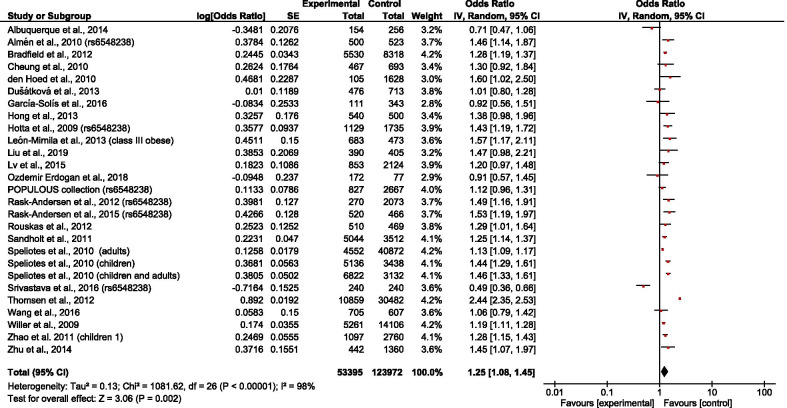
Forest plot of the association between rs6548238 polymorphisms (and its surrogates) near the TMEM18 gene and obesity risk. On the left are the name of the first author of the study and publication year. The horizontal line shows the corresponding 95% CI of the odds ratio (OR). The overall OR is based on the Generic Inverse Variance random effect model shown by the diamond. The solid vertical line represents the null result. Forest plot created within RevMan 5.3 software

**Fig. 3:**
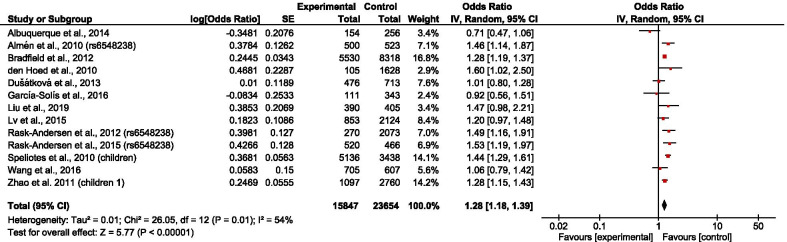
Forest plot of the association between child obesity and rs6548238 polymorphisms (and its surrogates) near gene TMEM18. On the left are the name of the first author of the study and publication year. The horizontal line shows the corresponding 95% CI of the odds ratio (OR). The overall OR is based on the Generic Inverse Variance method in random effect model shown by the diamond. The solid vertical line represents the null result. Forest plot created within RevMan 5.3 software

**Fig. 4:**
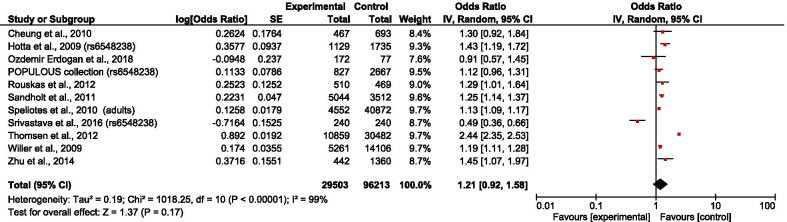
Forest plot of the association between adults obesity and rs6548238 polymorphisms (and its surrogates) near gene TMEM18. On the left are the name of the first author of the study and publication year. The horizontal line shows the corresponding 95% CI of the odds ratio (OR). The overall OR is based on the Generic Inverse Variance method in random effect model shown by the diamond. The solid vertical line represents the null result. Forest plot created within RevMan 5.3 software

**Fig. 5:**
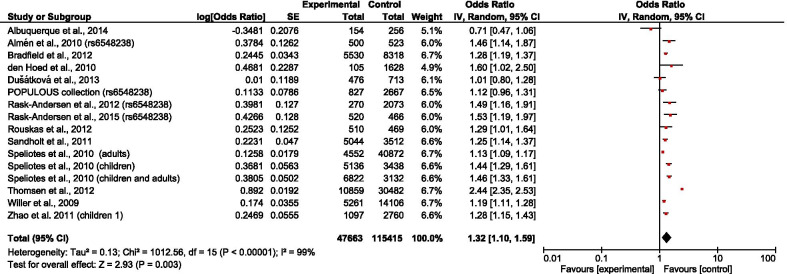
Forest plot of the association between European obesity and rs6548238 polymorphisms (and its surrogates) near gene TMEM18. On the left are the name of the first author of the study and publication year. The horizontal line shows the corresponding 95% CI of the odds ratio (OR). The overall OR is based on the Generic Inverse Variance method in random effect model shown by the diamond. The solid vertical line represents the null result. Forest plot created within RevMan 5.3 software

**Fig. 6:**
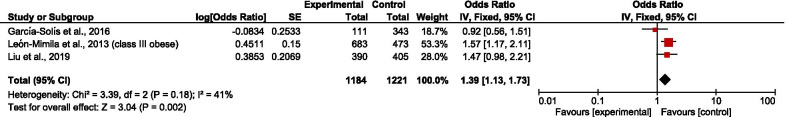
Forest plot of the association between Mexican obesity and rs6548238 polymorphisms (and its surrogates) near gene TMEM18. On the left are the name of the first author of the study and publication year. The horizontal line shows the corresponding 95% CI of the odds ratio (OR). The overall OR is based on the Generic Inverse Variance method in random effect model shown by the diamond. The solid vertical line represents the null result. Forest plot created within RevMan 5.3 software

**Fig. 7:**
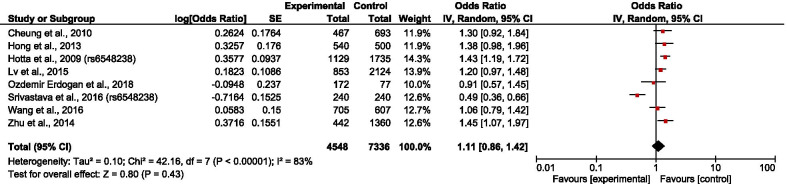
Forest plot of the association between Asian obesity and rs6548238 polymorphisms (and its surrogates) near gene TMEM18. On the left are the name of the first author of the study and publication year. The horizontal line shows the corresponding 95% CI of the odds ratio (OR). The overall OR is based on the Generic Inverse Variance method in random effect model shown by the diamond. The solid vertical line represents the null result. Forest plot created within RevMan 5.3 software

The overall results showed that rs6548238 and its surrogate was significantly associated with obesity risk (OR = 1.25; 95% CI: 1.08–1.45) (Fig. 2). In addition, a significant association was observed in children (OR = 1.28; 95% CI: 1.18–1.39) (Fig. 3) but not in adults (OR = 1.21; 95% CI: 0.92–1.58) (Fig. 4). In the subgroup stratified by population type, the effect size was significant among both European (OR = 1.32; 95% CI: 1.10–1.59) (Fig. 5) and Mexican (OR = 1.39; 95% CI: 1.13–1.73) (Fig. 6) but not in Asian populations (OR = 1.11; 95% CI: 0.86–1.42) (Fig. 7).

Based on 1000 Genomes Project data, polymorphism rs6548238 is used in the main analysis since it is in a high linkage disequilibrium with rs4854344 (D’ = 1; r = 1 in CEU (Utah Residents (CEPH) with Northern and Western European ancestry); D’ = 1; r = 1 in CHB (Han Chinese in Beijing, China); D’ = 1; r = 1 in MXL (Mexican Ancestry from Los Angeles USA), rs11127485 (D’ = 1; r = 1 in CEU; D’ = 1; r = 1 in CHB; D’ = 1, r = 1 in MXL), rs2867125 (D’ = 1; r = 0.964 in CEU; D’ = 1; r = 1 in CHB; D’ = 1; r = 1 in MXL) and rs7561317 (D’ = 1; r = 1 in CEU; D’ = 1; r = 1 in CHB; D’ = 1; r = 1 in MXL).

In the analysis of each polymorphism without division into subgroups, independently pooled ORs indicate that allele C of rs6548238 (OR = 1.19; 95% CI: 0.98–1.64) (Additional file 1: Fig. S1) was not related with the risk of obesity; however, some of the other analysed alleles, including allele T of rs4854344 (OR = 1.41; 95% CI: 1.24–1.60) (Additional file 2: Fig. S2) allele T of rs11127485 (OR = 1.49; 95% CI: 1.20–1.87) (Additional file 3: Fig. S3) and allele C of rs2867125 (OR = 1.29; 95% CI: 1.16–1.43) (Additional file 4: Fig. S4) were associated with the risk of obesity. The allele G of rs7561317 did not appear to demonstrate any significant relationship with obesity (OR 1.08; 95% CI: 0.88–1.32) (Additional file 5: Fig. S5).

### Publication bias and sensitivity analysis

**Fig. 8:**
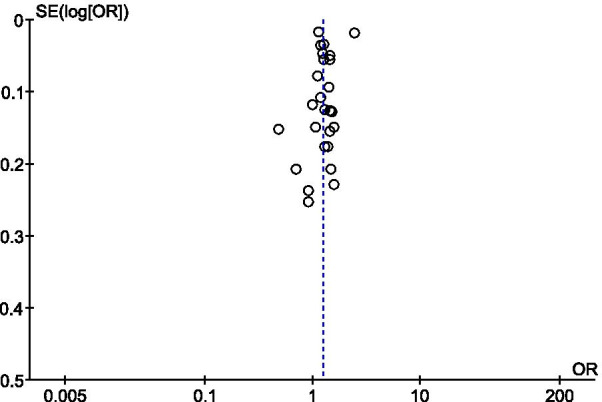
Funnel plot of Odds Ratio vs. Standard Error for log OR using a random effect model to evaluate publication bias for the effect of polymorphism rs6548238 and its proxy obesity. Funnel plot created within forest plot created using RevMan 5.3 software

Any publication bias regarding rs6548238 and its surrogates in the overall analysis was determined using funnel plots. The funnel plot revealed some asymmetry caused by the small study effects, or by the publication (Fig. 8).

To assess whether a single study could influence the main results, each study from the overall analysis was excluded. This allowed us to evaluate the influence of each study on the pooled effect size and the heterogeneity of the main results. However, the removal of any individual study from the data set did not substantially influence the OR in the overall analysis (Additional file 7: Table S2).

## Discussion

Studies examining the effect of genetics on obesity allowed to find new genetic factors involved in the pathogenesis of this disease. One of the studied regions was p25 of human chromosome 2, which is near the gene *TMEM18.* Some studies indicate a clear correlation between the polymorphisms found in this region and the risk of obesity [11, 12, 32, 45], while others indicate no significant changes [28, 33]. The present meta-analysis was performed to evaluate the association of SNPs near *TMEM18* with obesity with regard to age, i.e. children and adults, as well as population type. If *TMEM18* polymorphisms serve as risk factors for obesity, then it would be reasonable to assume that the alleles: C of rs6548238, T of rs4854344, T of rs11127485, C of rs2867125, G of rs7561317 will occur more often in obese people. To clarify these inconsistencies, the present meta-analysis was performed.

Most research has focused on the relationship between genetic factors and obesity-related traits among obese individuals, using lean individuals as a control group [17, 46, 47]. Many studies have carried out analyses involving the evaluation of BMI-increasing alleles with separation of ethnic [48] or age [49] groups. There is evidence that influence of genetic factors on the development of obesity differs among populations [50]. There is also evidence that the effects of common genetic variants on BMI profiles vary between life periods [51–54]. Obesity is a multifactorial and complex disease with a range of environmental, socioeconomic, behavioural and genetic influences: the significant increase in the number of obese people observed over the last few decades cannot be related solely to genetics. This suggests that environmental and behavioural factors are mostly responsible for obesity [55, 56]. Many studies have noted that the genetic contribution to BMI increases during childhood, but then decreases during adulthood, possibly due to the cumulative effects of many factors [57]. Adults are subject to a longer period of exposure to environmental, behavioural and socioeconomic factors which contribute to the development of obesity. The effect size of genetic factors which can predispose to obesity also differs between different populations across the world; such differenced may be associated with disparities in physiological responses to fat storage or different body shapes.

A key problem for the meta-analysis of genetic studies is presented by their frequent heterogeneity; this arises through various biases due to population stratification, misclassification of clinical outcome, genotyping errors and the overestimation of genetic effects in the original study, as well as a difference in the pattern of LD structure across populations [58]. In the present meta-analysis, greater heterogeneity was observed in the group of adults than the group of children; this difference is connected with the wider age range and the longer period of exposure to environmental and socioeconomic factors which affect obesity.

The strengths of the current study include the relatively large sample size from people around the world, the robustness of statistical analysis and additional data for meta-analysis (POPULOUS collection data). Despite all of these strengths, some limitations should be addressed. OR with 95% CI from genetic data was used to evaluate the sole genetic influence; however, when it was not given, calculated OR with 95% CI was used after covariate adjustment from the individual study. The potential interactions between the genetic factors and environmental factors were not addressed due to insufficient data, which might also affect the accuracy of the results.

The second limitation concerns the variation in BMI cut-offs for obesity between studies. Most studies used the basic classification of obesity (BMI ≥ 30) and normal weight (BMI 18.5–24.9); however, in some studies, mostly among Asian populations, other BMI thresholds were used for obesity (e.g. BMI ≥ 27.5) and normal weight (e.g. BMI 18.5–23.0) (Additional file 6: Table S1). It is possible that the authors customized these ranges to better fit them to a particular studied population. These differences suggest that these ranges may be dependent on population type, and as such, could differ between European, Asian or other populations. Therefore, their comparison may be not so obvious. This was true for both adults and children, because some studies have used customized values of BMI percentiles to define obesity or normal weight in children.

The present meta-analysis is the first to systematically explore the association between *TMEM18* polymorphisms and obesity across different population types based on ethnicity and age. Our findings indicate that rs6548238 was significantly associated with an increased risk of obesity and similar trends were found among subgroups of European and Mexican origin but not Asian. While the presence of this polymorphism appears to have a significant influence on obesity among children, no significant association was found with obesity risk among adults.

## Conclusions

The results of this meta-analysis suggest an association between rs6548238 polymorphisms and its surrogate near *TMEM18* and the risk of obesity. Differences appear to exist between ethnic populations and age groups. Our findings support those of a previous study showing the various effect of genetics in age and ethnic groups.

## Supplementary Information

**Additional file 1: Fig. S1**. Meta-analysis of the association between rs6548238 and obesity risk using random effect model. Forest plot created within RevMan 5.3 software.

**Additional file 2: Fig. S2**. Meta-analysis of the association between rs4854344 and obesity risk using random effect model. Forest plot created within RevMan 5.3 software.

**Additional file 3: Fig. S3**. Meta-analysis of the association between rs11127485 and obesity risk using fixed effect model. The size of the red box corresponding to each study is proportional to the sample size. Forest plot created within RevMan 5.3 software.

**Additional file 4: Fig. S4**. Meta-analysis of the association between rs2867125 and obesity risk using random effect model. Forest plot created within RevMan 5.3 software.

**Additional file 5: Fig. S5**. Meta-analysis of the association between rs7561317 and obesity risk using random effect model. Forest plot created within RevMan 5.3 software.

**Additional file 6: Table S1**. Characteristics of studies included in the meta-analysis.

**Additional file 7: Table S2**. Sensitivity analysis of general results of rs6548238 and its surrogates.

**Additional file 8: Table S3**. The quality of studies included in the analysis.

## Data Availability

Data sharing is not applicable to this article as no datasets were generated or analysed during the current study with the exclusion of the dataset from the POPULOUS study. To apply for permission and access to the raw data from POPULOUS study, please contact directly with corresponding author.

## Abbreviations

BBMRI-ERIC: Biobanking and Biomolecular Resources Research Infrastructure-European Research Infrastructure Consortium
BMI: Body Mass Index
CEU: Utah Residents (CEPH) with Northern and Western European ancestry, from 1000 Genome Project
CHB: Han Chinese in Beijing, China, from 1000 Genome Project
GWAS: Genome-wide Association Study
MXL: Mexican ancestry from Los Angeles USA, from 1000 Genome Project
OR: Odds ratio
TMEM18: Transmembrane protein 18
