# Supplementary figures and images for: The association between polymorphisms near TMEM18 and the risk of obesity: a meta-analysis
Source: BMC Med Genomics. 2021 Jul 6;14:179. doi: 10.1186/s12920-021-01025-7 (PMC8259011; doi:10.1186/s12920-021-01025-7)

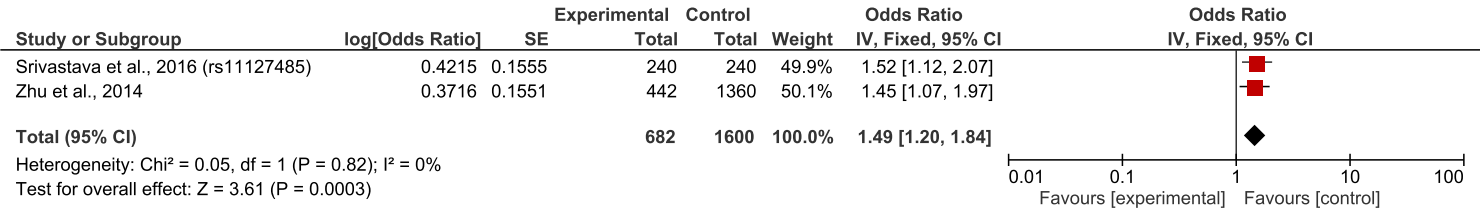

Supplement: Supplementary file 1 — Additional file 1: Fig. S1. Meta-analysis of the association between rs6548238 and obesity risk using random effect model. Forest plot created within RevMan 5.3 software. [file 12920_2021_1025_MOESM1_ESM.pdf]

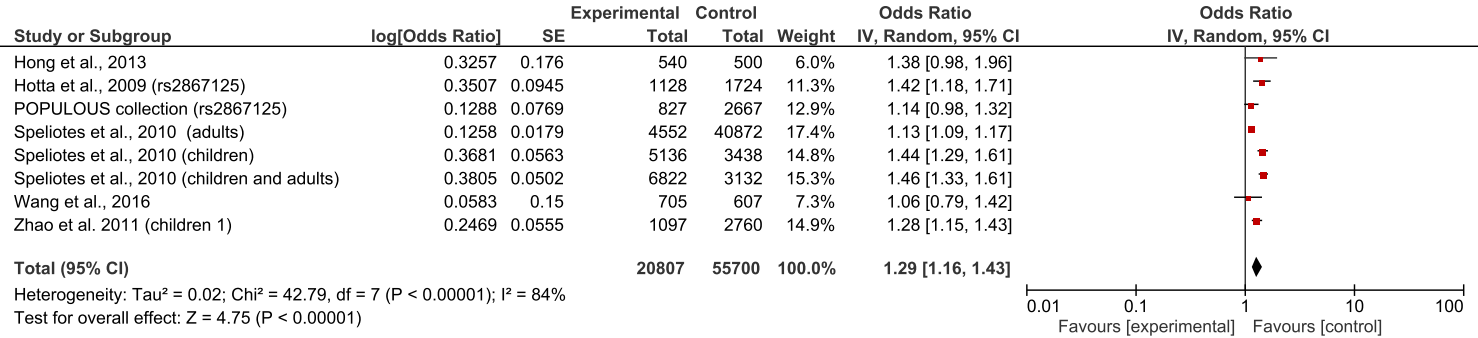

Supplement: Supplementary file 2 — Additional file 2: Fig. S2. Meta-analysis of the association between rs4854344 and obesity risk using random effect model. Forest plot created within RevMan 5.3 software. [file 12920_2021_1025_MOESM2_ESM.pdf]

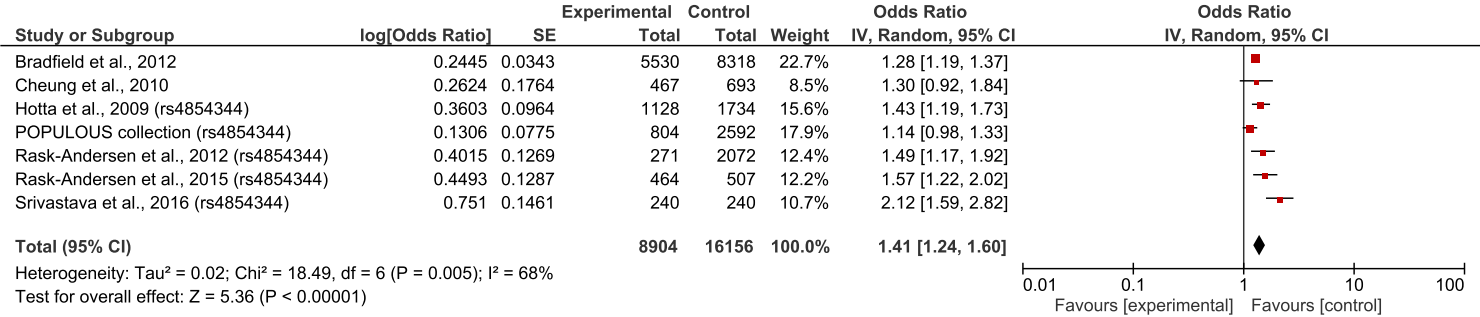

Supplement: Supplementary file 3 — Additional file 3: Fig. S3. Meta-analysis of the association between rs11127485 and obesity risk using fixed effect model. The size of the red box corresponding to each study is proportional to the sample size. Forest plot created within RevMan 5.3 software. [file 12920_2021_1025_MOESM3_ESM.pdf]

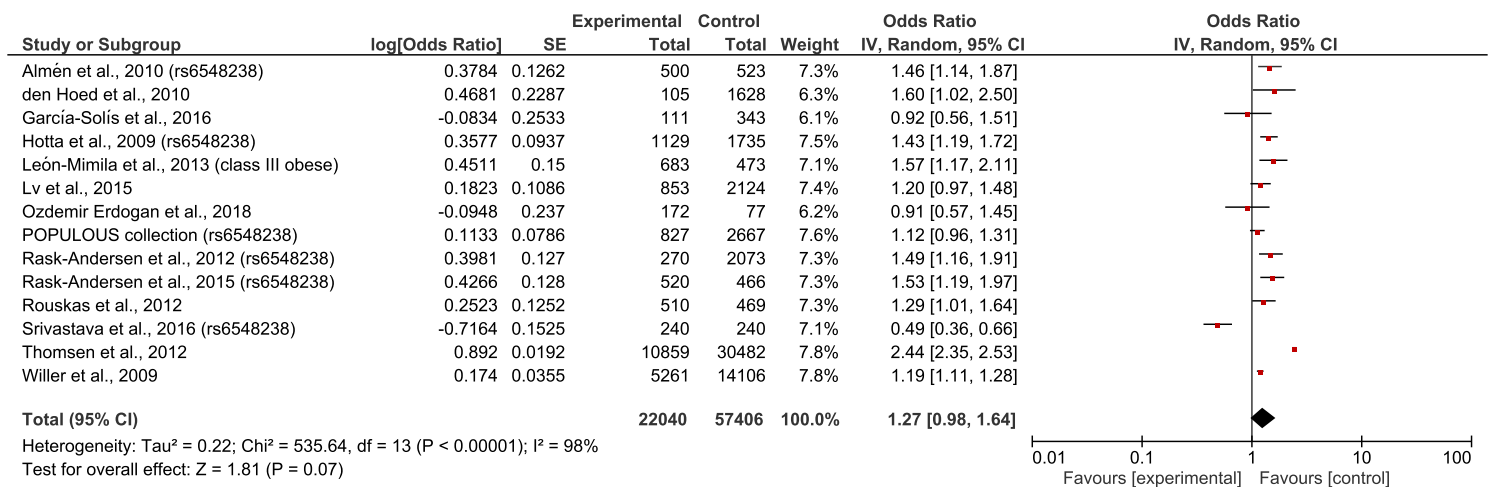

Supplement: Supplementary file 4 — Additional file 4: Fig. S4. Meta-analysis of the association between rs2867125 and obesity risk using random effect model. Forest plot created within RevMan 5.3 software. [file 12920_2021_1025_MOESM4_ESM.pdf]

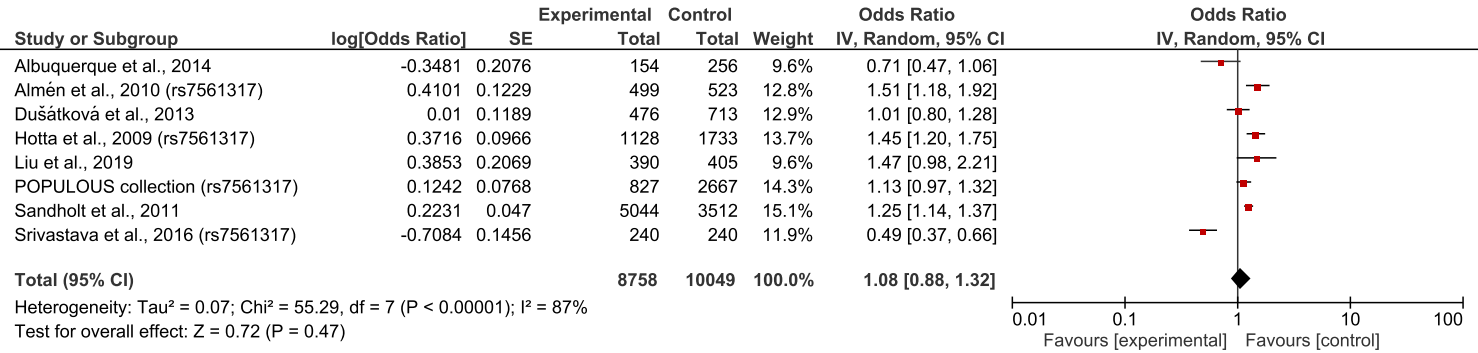

Supplement: Supplementary file 5 — Additional file 5: Fig. S5. Meta-analysis of the association between rs7561317 and obesity risk using random effect model. Forest plot created within RevMan 5.3 software. [file 12920_2021_1025_MOESM5_ESM.pdf]
